# Single-cell profiling of PBMCS reveals an immune signature of irAEs in anti-PD-1-treated acral melanoma patients

**DOI:** 10.3389/fimmu.2026.1758205

**Published:** 2026-02-26

**Authors:** Qi Bao, Jie Pan, Zhefang Wang

**Affiliations:** 1 Department of Plastic and Reconstructive Surgery, Second Affiliated Hospital of Zhejiang University School of Medicine, Hangzhou, China; 2 Department of Endocrinology and Metabolism, The Second Affiliated Hospital Zhejiang University School of Medicine, Hangzhou, China

**Keywords:** acral melanoma, anti-PD-1 therapy, cytotoxic CD8+ T cells, GZMK, immune-related adverse events (irAEs)

## Abstract

**Introduction:**

Immune checkpoint inhibitors (ICIs) targeting PD-1 have revolutionized melanoma treatment, yet their clinical efficacy is frequently limited by immune-related adverse events (irAEs). The underlying mechanisms of irAEs remain poorly defined, particularly in the acral melanoma subtype.

**Methods:**

To identify peripheral immune signatures associated with irAE development, we performed single-cell RNA sequencing (scRNA-seq) on peripheral blood mononuclear cells (PBMCs) from eight acral melanoma patients: three who developed irAEs on anti-PD-1 therapy (AE), three treated patients without irAEs (NAE), and two untreated controls (UNT). Cellular composition, transcriptional profiles, and differentiation trajectories were analyzed.

**Results:**

Analysis of 54,793 high-quality cells revealed a profound reconfiguration of the CD8+ T cell compartment specifically in AE patients. This was characterized by an expansion of cytotoxic CD8+ T cells (enriched for *GZMB*, *GNLY*, *NKG7*) and a concurrent contraction of a transitional CD8+ T cell population marked by *GZMK* expression. Consequently, the ratio of transitional to cytotoxic CD8+ T cells was decreased in the AE group. Pseudotime trajectory analysis confirmed that GZMK+ transitional cells represent an intermediate differentiation state between naïve and terminal cytotoxic phenotypes. Additionally, AE patients exhibited an elevated proportion of proliferating T cells and enrichment of cell-killing gene pathways.

**Discussion:**

Our findings propose a model wherein an imbalance in CD8+ T cell differentiation, favoring aggressive cytotoxic effectors over a putative buffering transitional population, underpins irAEs pathogenesis in acral melanoma patients receiving anti-PD-1 therapy. The transitional-to-cytotoxic CD8+ T cell ratio emerges as an exploratory candidate biomarker for irAEs risk, warranting validation in larger prospective cohorts.

## Introduction

The management of advanced melanoma has been revolutionized by immune checkpoint inhibitors (ICIs), which block regulatory pathways such as PD-1 to reinvigorate antitumor T-cell immunity. However, the enhanced immune activation driven by ICIs frequently leads to immune-related adverse events (irAEs), which affect a broad range of organs and can range from mild and self-limiting to severe, life-threatening complications (1–3). For patients receiving anti-PD-1 monotherapy, irAEs most commonly involve skin, endocrine glands, and gastrointestinal system of melanoma patients (4). irAEs usually emerge within several weeks to months after the initiation of ICI and most of them can be treated with immunosuppressive agents such as glucocorticoids (5). While often manageable with immunosuppressants, irAEs represent a major clinical challenge that can necessitate treatment discontinuation and compromise patient quality of life.

The pathophysiological mechanisms underlying irAEs remain incompletely elucidated but are thought to share similarities with autoimmune disorders (6, 7). Proposed mechanisms include cross-reactivity between tumor and self-antigens (8, 9), aberrant B-cell and auto-antibody activity (10), inflammatory cytokine release (11–13), and alterations in the gut microbiome (14). Despite these insights, predictive biomarkers for irAEs are lacking, and the specific immune cell subsets and states that predispose patients to these toxicities are poorly defined, particularly at the single-cell level. This gap in knowledge is especially pronounced in acral melanoma, a subtype with distinct clinicopathological features and a lower mutational burden that may respond differently to immunotherapy (15). Furthermore, while numerous studies have focused on tissue-resident immunity, peripheral blood mononuclear cells (PBMCs) offer a minimally invasive window into systemic immune activation. PBMCs dynamically reflect the immune response to ICIs and harbor tumor-reactive T cells (16), making them a valuable resource for identifying circulating biomarkers of both efficacy and toxicity. Previous studies have suggested associations between irAEs and various immune populations, including CD16+ monocytes (17), CD4 memory T cells (6), CD8 T cells (18, 19), MAIT (Mucosal-associated invariant T cells) (9) and Tregs (18). However, these findings are often inconsistent across cohorts, and the functional characteristics of these cells remain unexplored. A high-resolution understanding of the peripheral immune landscape is therefore critical to deciphering the basis of irAE development.

In this study, we performed single-cell RNA sequencing (scRNA-seq) on PBMCs from acral melanoma patients receiving anti-PD-1 monotherapy (primarily toripalimab) to comprehensively characterize the immune signatures associated with irAEs. We identified a profound shift within the CD8+ T-cell compartment, specifically an expansion of cytotoxic cells and a contraction of a transitional GZMK+ population, in patients experiencing irAEs. Our results delineate a potential mechanism for irAE pathogenesis and propose a novel cellular ratio as an exploratory candidate biomarker for immune toxicity.

## Results

### Patient characteristics and irAEs

The characteristics of eight melanoma patients, including three who developed irAEs (AE group) following ICI therapy, three who did not develop irAEs (NAE group) post-ICI therapy, and two control patients who did not receive ICI therapy (UNT group), are summarized in **Table 1**. The specific irAEs observed included nausea, vomiting and fatigue in one patient after eight cycles of pembrolizumab, skin rash and vitiligo in another after four cycles of toripalimab, and erythematous papules in a third patient after two cycles of toripalimab. Patients in non-irAEs group were all treated with toripalimab.

**Table 1:** Characteristics of patients.

No.	Age	Sex	Tumor site	Tumor stage	Treatment	IrAE	Grouping
1	84	M	Foot	II	None	No	UNT
2	68	F	Foot	II	None	No	UNT
3	61	F	Hand	II	pembrolizumab	Pituitary	AE
4	75	M	Foot	III	toripalimab	Skin	AE
5	63	M	Foot	III	toripalimab	Skin	AE
6	79	M	Foot	III	toripalimab	No	NAE
7	47	M	Foot	II	toripalimab	No	NAE
8	70	M	Foot	III	toripalimab	No	NAE

### Anti-PD-1 therapy induces a systemic shift in PBMC composition

**Figure 1:**
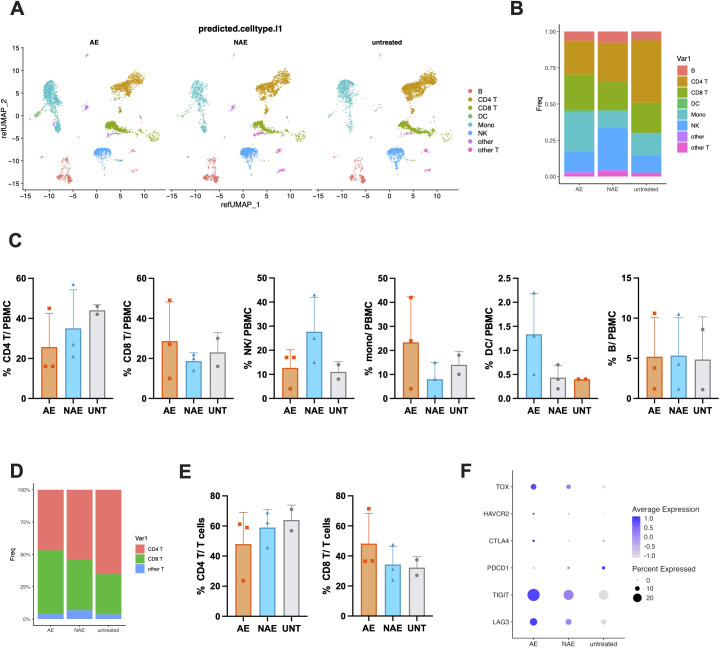
Anti-PD-1 therapy induces a systemic shift in PBMC composition. **(A)** UMAP projection of 54,793 high-quality peripheral blood mononuclear cells (PBMCs) from all eight patients, split by group, colored by major cell type clusters. **(B)** Stacked bar chart illustrate the distribution of major immune cell types across the three patient groups: untreated (UNT), treated without irAEs (NAE), and treated with irAEs (AE). 24042, 22842, and 7909 cells were included in AE, NAE, and UNT groups, respectively. Data represent pooled samples within each group. **(C)** Bar charts comparing the frequency of major immune cell types (CD4 T, CD8 T, NK, Monocyte, DC, B cells) among the AE (n=2), NAE (n=3), and UNT (n=3) groups. Data points represent individual patients, with bars showing the mean ± SD. **(D, E)**. Stacked bar chart and bar chart of CD4 T and CD8 T cells normalized to all T cells. **(F)** Dot plot showing the expression level (average expression and percent expressed) of key immune checkpoint genes (PDCD1, CTLA4, TIGIT, LAG3, HAVCR2) and the transcription factor TOX in T cells.

Single-cell RNA sequencing (scRNA-seq) was performed on peripheral blood mononuclear cells (PBMCs) to assess the composition and functional changes of various immune cells in melanoma patients undergoing anti-PD-1 therapy. After excluding low-quality cells, a total of 54,793 cells were analyzed and mapped to a multimodal PBMC reference dataset, and the results were visualized using Uniform Manifold Approximation and Projection (UMAP) plots (**Figure 1A**). The relative abundance of these broad immune cell categories across different patient groups is depicted in **Figure 1B**. We observed substantial alterations in the immune cell landscape: there was a decrease in the frequency of CD4 T cells and an increase in CD8 T cells in the AE group compared to both the NAE group and the UNT group (**Figure 1C**). Notably, the NAE group exhibited a higher frequency of NK cells and a lower frequency of monocytes than either the AE group or the UNT group (**Figure 1C**). Meanwhile, the frequency of DC was slightly higher in AE group, whereas the frequency of B cells remained comparable across all groups (**Figure 1C**). When the data were normalized to total T cells, the frequency of CD8 T cells was found to be highest in the AE group and lowest in the UNT group (**Figures 1D, E**). Conversely, the frequency of CD4 T cells was lowest in the AE group and highest in the UNT group, with the NAE group displaying intermediate frequencies (**Figures 1D, E**). To further assess the impact of anti-PD-1 treatment, we examined the expression levels of PD-1 and other immune checkpoint receptors. A dot plot analysis revealed a decreased expression of PDCD1 (PD-1) and an increased expression of CTLA4, TIGIT, LAG3, HAVCR2 (TIM-3), and the transcriptional factor TOX in T cells post-treatment compared to the UNT group (**Figure 1F**). These changes indicate the effectiveness of anti-PD-1 therapy in both the AE and NAE groups and suggest a compensatory regulation of immune checkpoint receptors during treatment, with these effects being more pronounced in the AE group. In summary, anti-PD-1 therapy was associated with a shift in the balance of T cell subpopulations within PBMCs, characterized by an increased frequency of CD8 T cells and a reduced frequency of CD4 T cells. This shift was most pronounced in the AE group, underscoring the potential link between T cell dynamics and the development of irAEs during therapy.

### Expansion of cytotoxic and proliferating T cells distinguishes patients with irAEs

**Figure 2:**
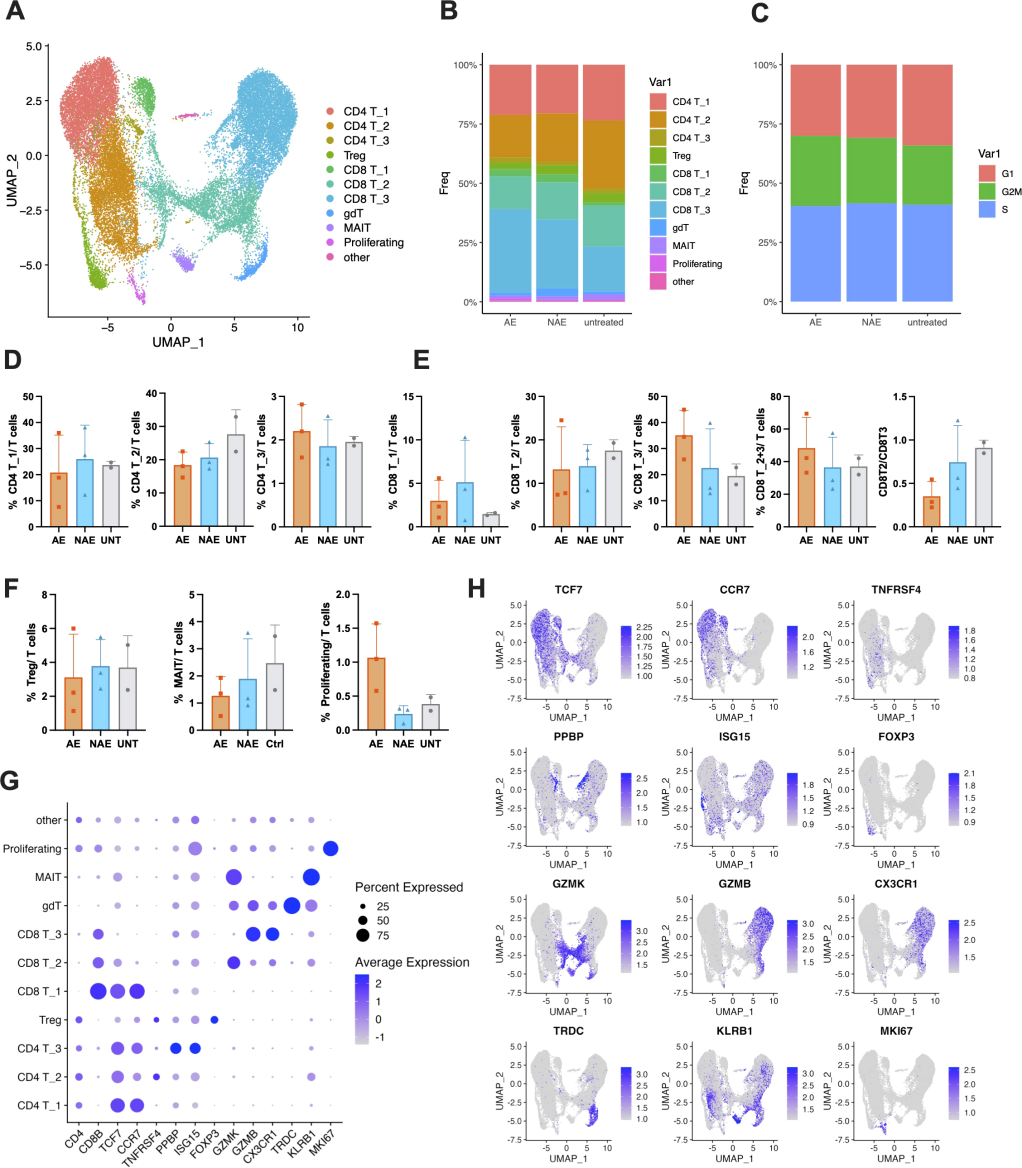
Expansion of cytotoxic and proliferating T cells distinguishes patients with irAEs. **(A)** UMAP projection of all T cells (28512 cells), colored by 11 distinct T cell subclusters. **(B, C)**. Stacked bar chart illustrate the distribution of T cell subclusters **(B)** and cell cycle phases **(C)** across groups. 11949, 11351, and 5212 cells were included in AE, NAE, and UNT groups, respectively. Data represent pooled samples within each group. **(D-F)**. Bar chart comparing the frequency of main clusters of T cells among the AE, NAE, and UNT groups. Data points represent individual patients, with bars showing the mean ± SD. G-H. **(G)** Dot plot and **(H)** feature plots visualizing the expression of canonical marker genes used to define distinct T cell subclusters.

To determine the specific T cell subsets associated with the development of adverse events in melanoma patients undergoing anti-PD-1 therapy, all T cells were extracted for sub-analysis. UMAP visualization categorized T cells into 11 distinct clusters: three CD4 T cell clusters, three CD8 T cell clusters, T regulatory cells (Tregs), gamma delta T cells (gdT), Mucosal-associated invariant T cells (MAIT), proliferating T cells, and other unspecified T cells (**Figure 2A**). Using canonical markers, we defined CD4 T_1 and CD8 T_1 as naïve T cells (TCF7+), CD4 T_2 as activated memory CD 4+ T cells (TNFRSF4+), CD8 T_2 as effector-memory-like CD8+ T cells (GZMK+), and CD8 T_3 as terminal effector/cytotoxic CD8+ T cells (CX3CR1+). The CD4 T_3 cluster displayed a distinct profile, co-expressing platelet-related genes (e.g., PPBP, PF4) and a strong interferon-stimulated gene signature (e.g., ISG15, IFIT1). This pattern may reflect a state of tumor-educated, hyperactivated CD4+ T cells, though a contribution from potential cell doublets cannot be excluded. The expression and distribution of these markers are visualized as DotPlot and FeaturePlot in **Figures 2G, H**. The relative abundance and cell cycle distribution of each cluster were analyzed (**Figures 2B, C**). Notably, the AE group exhibited a slightly higher percentage of cells in the G2/M phase, indicating enhanced cellular proliferation. Consistently, the frequency of proliferating T cells was increased in the AE group compared to NAE group (**Figure 2F**). While overall T cell cycle activity shows a trend in NAE group, the clonal expansion of a dedicated proliferating T cell population is a distinctive feature of the AE group. Following anti-PD-1 therapy, the frequency of CD4 T_2 and CD8 T_2 cells decreased, while the frequency of CD8 T_3 cells increased in the AE group compared to both the untreated and NAE groups (**Figures 2D, E**). Meanwhile, the combined effector CD8 T cells (CD8 T_2 and CD8 T_3) were increased in AE group (**Figure 2E**). However, these changes did not reach statistical significance, likely due to the limited sample size. Though not statistically significant, the ratio of CD8 T_2 to CD8 T_3 cells was considerably decreased in the AE group compared to the UNT group (**Figure 2E**). Additionally, the frequencies of CD4_1 cells and Treg cells remained unchanged across all groups (**Figures 2D, F**). In summary, the analysis revealed that the frequency of CD8 T_3 (cytotoxic T) cells and proliferating T cells were elevated in the AE group. This pattern suggests a mechanistic link between the enhanced propensity of T cells toward a cytotoxic and proliferative phenotype and the development of irAEs in patients receiving anti-PD-1 therapy.

### Cytotoxic and GZMK+ transitional gene programs define divergent CD8+ T cell states

**Figure 3:**
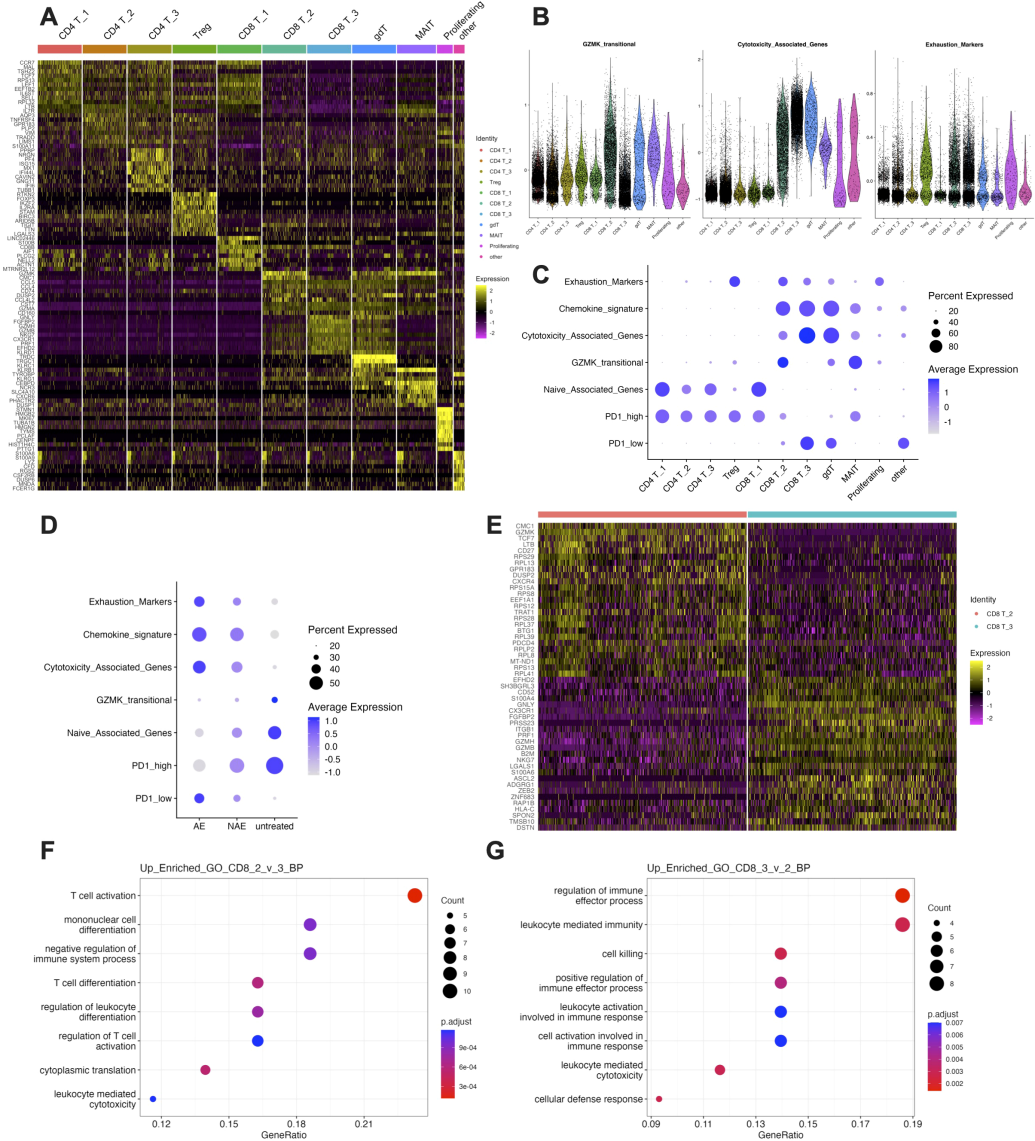
Cytotoxic and GZMK+ transitional gene programs define divergent CD8+ T cell states. **(A)** Heatmap of the top 10 differentially expressed genes (DEGs) for each of the 11 T cell clusters. **(B)** Violin plots showing the Gene Set Variation Analysis (GSVA) scores for GZMK-transitional and cytotoxicity signatures across all T cell clusters. C-D. Dot plot visualizing the GSVA scores for the exhaustion, chemokine, cytotoxicity, GZMK-transitional, naïve, PD-1 high, and PD-1 low gene signatures across **(C)** all T cell clusters and **(D)** patient groups. **(E)** Heatmap of the top 25 DEGs between the CD8 T_2 (transitional) and CD8 T_3 (cytotoxic) clusters. **(F, G)**. Gene Ontology (GO) biological process terms significantly enriched in the genes upregulated in **(F)** CD8 T_2 cluster and **(G)** CD8 T_3 cluster.

To further delineate the molecular characteristics of each T cell cluster, differentially expressed genes (DEGs) were calculated using FindAllMarkers function and heatmap was plotted with top 10 genes in each cluster (**Figure 3A**). The CD8 T_3 cluster demonstrated enrichment of cytotoxic T cell markers, including GNLY, FGFBP2, GZMH, GZMB, NKG7, and CX3CR1, indicating a terminally differentiated cytotoxic phenotype. These markers were also present in the CD8 T_2 cluster, albeit at lower levels (**Figure 3A**). Notably, CD8 T_2 cluster was marked by GZMK, CCL4, CCL5, DUSP2, and CD160, indicating a effector memory phenotype. To assess the functionality of these clusters, Gene Set Variation Analysis (GSVA) scores for pathways related to immunotherapy were calculated. Both CD8 T_2 and CD8 T_3 clusters displayed elevated cytotoxic scores compared to other clusters, with CD8 T_3 exhibiting the highest scores (**Figures 3B, C**). In contrast, CD8 T_2 was marked by the highest expression of GZMK+ transitional genes (PMID: 33271118), which was markedly lower in CD8 T_3 (**Figures 3B, C**). Notably, the exhaustion score was similar between CD8 T_2 and CD8 T_3. Additionally, both the PD-1 high and PD-1 low signatures were modestly enriched in CD8 T_2. However, CD8 T_3 exhibited the highest PD-1 low signature and the lowest PD-1 high signature, suggesting that CD8 T_3 was the primary cluster affected following anti-PD-1 therapy (**Figure 3C**). Upon comparing treatment groups, it was evident that anti-PD-1 therapy enhanced the expression of cytotoxicity and exhaustion-related pathways while diminishing the expression of pathways associated with GZMK+ transitional genes, naïve cell markers, and high PD-1 expression (**Figure 3D**). Given the established link between GZMK and immune aging (20), we assessed its correlation with chronological age in our cohort. Analysis revealed no significant association between patient age and either GZMK expression or the GSVA score for the GZMK-transitional gene signature in T cells (**Supplementary Figures 1A, B**). To further examine the biological differences between CD8 T_2 and CD8 T_3, additional DEGs were identified and the top 25 genes in each cluster were displayed in a heatmap (**Figure 3E**). Subsequent gene ontology (GO) analysis revealed that the CD8 T_2 was more enriched in pathways associated with T cell activation and differentiation (**Figure 3F**), whereas the CD8 T_3 was predominantly enriched in pathways linked to cytotoxicity (**Figure 3G**). Taken together, our findings highlight distinct molecular and functional profiles within the CD8 T cell subpopulations in melanoma patients receiving anti-PD-1 therapy.

### A diminished transitional-to-cytotoxic CD8+ T cell ratio is a hallmark of irAEs

**Figure 4:**
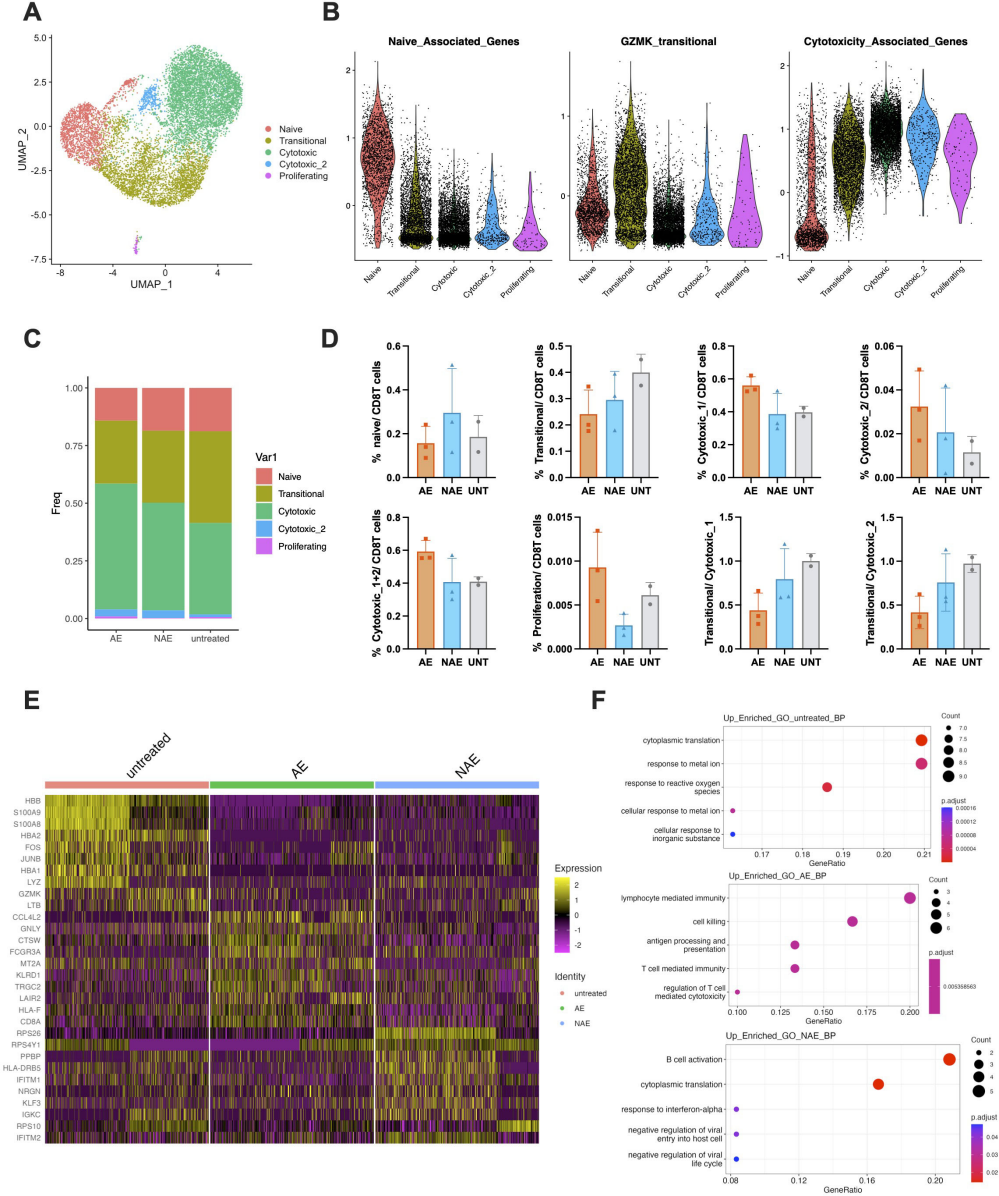
A diminished transitional-to-cytotoxic CD8+ T cell ratio is a hallmark of irAEs. **(A)** UMAP projection of all CD8 T cells (12014 cells), re-clustered and annotated into five clusters: naïve, transitional, cytotoxic, cytotoxic_2, and proliferating. **(B)** Violin plots showing the distribution of GSVA scores for naïve, cytotoxicity, and exhaustion signatures across the CD8 T cell states defined in **(A, C)** Stacked bar chart illustrate the distribution of CD8 T cell subclusters across the groups. 5901, 4486, and 1627 cells were included in AE, NAE, and UNT groups, respectively. Data represent pooled samples within each group. **(D)** Bar chart comparing the frequency of naïve, transitional, cytotoxic, cytotoxic_2, and proliferating cells normalized to all CD8 T cells across the groups. The ratio of transitional to cytotoxic CD8 T cells was plotted on the right. **(E)** Heatmap of the top 10 DEGs for the CD8 T cells across groups. **(F)** Gene Ontology (GO) biological process terms significantly enriched in the DEGs of the UNT (upper), AE (middle), and NAE (lower) groups compared to the others.

To gain a deeper understanding of immune changes in CD8 T cells during anti-PD-1 therapy, all CD8 T cells were extracted for further analysis. UMAP visualization categorized these cells into five clusters, naïve, transitional (GZMK+), cytotoxic_1, cytotoxic_2, and proliferating, the former three corresponding to CD8 T_1, CD8 T_2, and CD8 T_3 in **Figures 2A, respectively** (**Figure 4A**). Violin plots displaying GSVA scores revealed that transitional (GZMK+) cluster represents an intermediate state, showing moderate expression of naïve and cytotoxic signatures and a peak in the GZMK-transitional signature (**Figure 4B**). Following anti-PD-1 therapy, a substantial increase in cytotoxic_1 CD8+ T cells and a corresponding decrease in naïve and transitional (GZMK+) cells were observed in the AE group, meanwhile the low-frequency cytotoxic_2 subset followed a similar trend with cytotoxic_1 subset. However, these changes did not reach statistical significance due to the limited sample size (**Figures 4C, D**). Additionally, the ratio of transitional-to-cytotoxic T cells was considerably decreased in the AE group compared to the UNT group (**Figure 4D**). In contrast, the NAE group showed a slightly higher percentage of naïve CD8 T cells and a comparable percentage of cytotoxic cells relative to the UNT group (**Figure 4D**). Additionally, the AE group demonstrated increased proliferative capacity compared to both the NAE and UNT groups (**Figure 4D**). To delineate differences among the groups, DEGs were identified and the top 10 genes in each group were visualized through a heatmap (**Figure 4E**). Further GO analysis showed that the AE group was specifically enriched in cell-killing-associated pathways, while the UNT and NAE groups were more associated with metabolic and cell-activation pathways (**Figure 4F**). In summary, these results further indicate a marked increase in cytotoxic CD8+ T cells in the AE group following anti-PD-1 treatment, accompanied by a decreased ratio of transitional to cytotoxic CD8 T cells.

### Trajectory analysis delineates the differentiation path from naïve to cytotoxic CD8+ T cells

**Figure 5:**
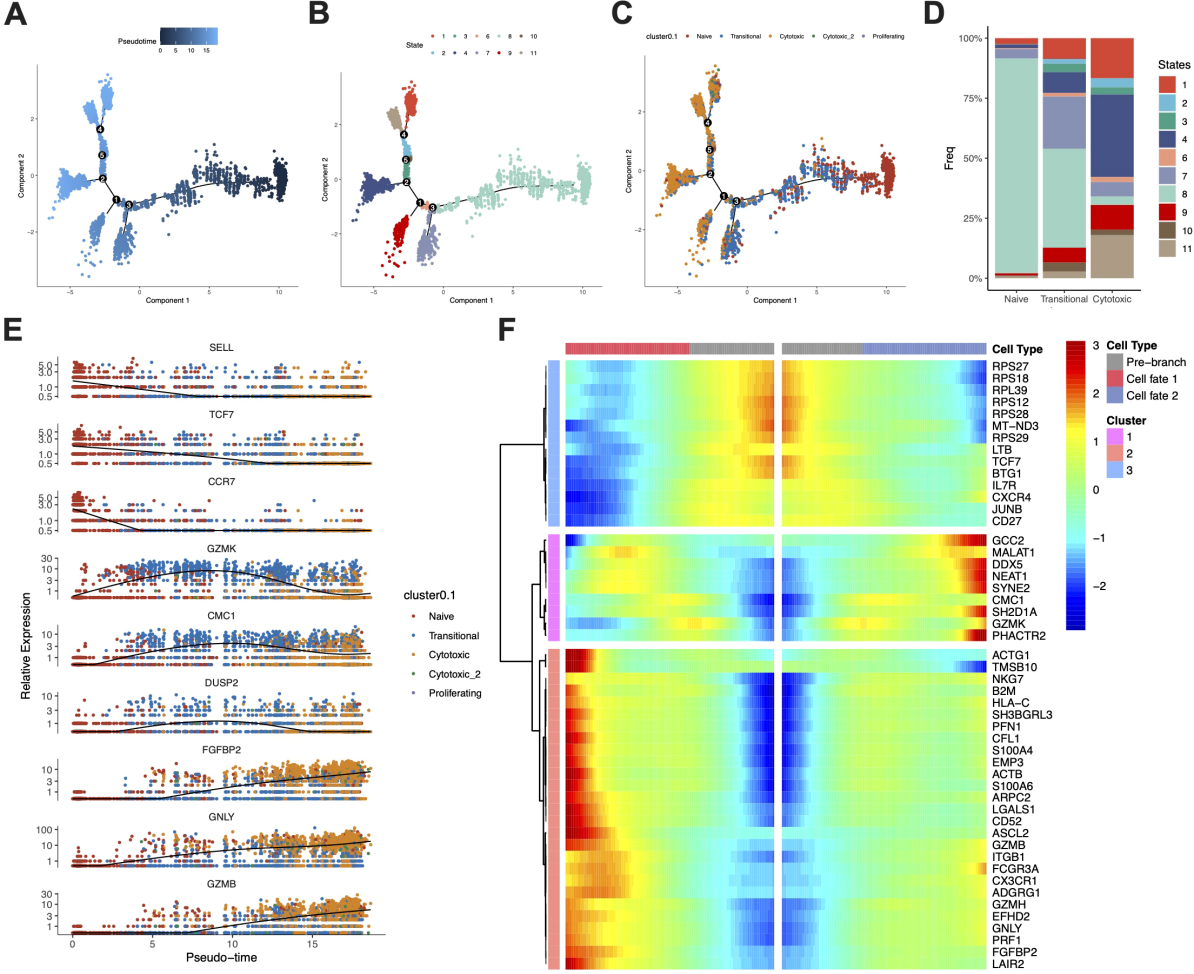
Trajectory analysis delineates the differentiation path from naïve to cytotoxic CD8+ T cells. **(A-C)**. Pseudotime trajectory of CD8 T cells, with cells colored by their **(A)** inferred pseudotime value, **(B)** states, and **(C)** assigned clusters **(D)** Stacked bar chart showing the distribution of all identified states across CD8 T cell clusters (Naive, Transitional, Cytotoxic). **(E)** Pseudotime kinetics of key marker genes over the inferred pseudotime trajectory. **(F)** Heatmap of genes that are differentially expressed along the two branching fates (towards transitional or cytotoxic cells) in the trajectory, as identified by Branch Expression Analysis Modeling (BEAM).

To elucidate the dynamics of CD8 T cell subpopulations during anti-PD-1 therapy, we conducted a trajectory analysis on CD8 T cells. This analysis identified two principal differentiation pathways originating from naïve CD8 T cells: one leading to transitional CD8 T cells and the other progressing to terminal cytotoxic CD8 T cells, as depicted in **Figures 5A–C**. Transitional CD8 T cells are predominantly located at the midpoint of this trajectory, indicating a crucial transitional phase prior to their full differentiation into the cytotoxic phenotype. Distribution analysis of these cells across the identified states revealed that naïve CD8 T cells were primarily found in state 8, whereas transitional CD8 T cells were concentrated in states 7 and 8, and cytotoxic CD8 T cells were distributed across other states, as shown in **Figure 5D**. To visualize the dynamic shifts in gene expression associated with these subpopulations, we plotted the expression levels of key genes over the pseudotime continuum in **Figure 5E**. Here, expression of naïve and cytotoxic markers peaked at the respective ends of the pseudotime axis, while markers such as GZMK, CMC1 and DUSP2 exhibited peaks during the transitional phase, suggesting a gradual shift in cellular function. Further refinement of this analysis was achieved through BEAM (Branch Expression Analysis Modeling), which identified genes differentially expressed along these differentiation paths. The cell fate toward transitional CD8 T cells was characterized by enrichment of genes such as MALAT1, NEAT1, SYNE2, CMC1, SH2D1A, and GZMK (**Figure 5F**), suggests a state of cellular preparation for a functional shift. Conversely, the trajectory toward cytotoxic CD8 T cells was characterized by enrichment of genes such as NKG7, GZMB, GNLY, PRF1, and FGFBP2 (**Figure 5F**), which are well-known for their roles in cell-mediated cytotoxicity. These findings indicate that transitional CD8 T cells may serve as a versatile intermediary, capable of modulating the immune response following anti-PD-1 therapy. This modulation may influence the balance between a regulated immune response and an aggressive cytotoxic attack, potentially mitigating or exacerbating the risks of irAEs.

## Discussion

The advent of ICIs has revolutionized melanoma treatment, yet their efficacy is accompanied by irAEs, which pose a significant clinical challenge and remain poorly predictable (5). While several molecular biomarkers have been linked to irAE risk, including cytokines (11, 21) (e.g. TNF, CXCL10), post-treatment neutrophil-to-lymphocyte ratio (NLR) (22), and activated T cell subsets or TCR diversity (23–25), their utility is limited by inconsistency across studies. In this pilot study, we performed scRNA-seq analysis using PBMC samples from acral melanoma patients who underwent anti-PD-1 therapy. Six patients received adjuvant anti-PD-1 therapy and two control patients were under observation. Of these six, five received toripalimab and one received pembrolizumab. Toripalimab is a recombinant humanized monoclonal antibody targeting programmed death receptor-1 (PD-1), which is being developed by Shanghai Junshi Bioscience Co., Ltd. in China for the treatment of various cancers (26, 27). To our knowledge, this study represents the first single-cell profiling of peripheral immune dynamics in acral melanoma patients under toripalimab treatment.

In consistent with literature, we found reduction of total CD4 T cells and elevation of total CD8 T cells and monocytes in patients with irAEs (AE) compared to patients without irAEs (NAE) and untreated patients (UNT) (17) (18). However, due to limited sample size, the difference did not show statistical significance. Further analysis of all T cells revealed distinct T cell dynamics in AE group versus NAE and UNT group. Notably, patients in the AE group exhibited increased cytotoxic CD8 T Cells, decreased GZMK+ transitional CD8 T Cells, and elevated proliferating cells. However, the reported reduction of MAIT and Tregs in patients with irAEs is not identified in our study (9, 18, 28).

Characterized by high expression of GZMB, PRF1, and GNLY, these cytotoxic CD8 T cells exhibit potent effector function, which, while beneficial for robust antitumor activity, may also suggests a propensity for off-target tissue damage leading to irAEs (29, 30). Notably, cytotoxic CD8 T cell frequencies remained unchanged in the NAE group, implying that their expansion is not merely a treatment artifact but rather a hallmark of irAE-prone immune states. Conversely, transitional CD8 T cells, marked by GZMK expression, appear to represent an intermediate stage between naïve and fully cytotoxic states. Although not statistically significant, the ratio of transitional-to-cytotoxic CD8+ T cells show the potential as exploratory candidate biomarker. These cells, enriched with GZMK+ transitional markers, suggest a more restrained cytotoxic capacity, as indicated by their lower cytotoxic GSVA score. This moderation may help temper aggressive immune responses, potentially balancing effective tumor control with the mitigation of therapy-related toxicity. Their depletion in AE patients could disrupt this balance, favoring unchecked cytotoxic activity and tissue injury. Additionally, trajectory analysis demonstrated that GZMK+ T cells occupy a transitional state along the naïve-to-cytotoxic differentiation continuum, further supporting their role as an intermediate phase in T cell maturation and activation. The differential expression of GZMK among CD8 T cell subsets and its association with transitional state provides a potential biomarker for predicting the emergence of irAE under anti-PD-1 therapy in melanoma patients, alongside the cytotoxic CD8 T cells. This highlights the transitional-to-cytotoxic CD8+ T cell ratio as an exploratory candidate biomarker for irAE risk, though further validation in larger cohorts is needed.

While GZMB has been extensively studied for its role in inducing cell death in target cells, GZMK has received comparatively less attention. Recent studies have linked GZMK expression to effector memory CD8 T cells by single-cell RNA sequencing analysis (31, 32). Supportively, one study identified a transitional T cell subtype (CD8+ TEM_GZMK_PDCD1) in PBMCs, which may be associated with a prolonged ICI response in NSCLC patients (33). Furthermore, another study reported a subtype of effector memory CD8 T cells featured with EMOS, GZMK and IFNG expression, highlighting their attenuated yet potential cytotoxic activity in lung cancer patients undergoing ICI treatment (34). However, the dynamics of this subtype appear to be dependent on specific types of irAEs. It would be intriguing to investigate whether our T cell subtype features, particularly the transitional-to-cytotoxic CD8 T cell ratio, also vary based on specific types of irAEs. However, due to the limited sample size in our study, we were unable to perform this analysis. Similarly, the transitional role of GZMK in tumor-infiltrating lymphocytes (TILs) was highlighted in a study investigating intra-tumoral changes during anti-PD-1 treatment in breast cancer patients (35). In which, GZMK was recognized as a marker of less differentiated, pre-effector T cells, showing peak expression halfway through the trajectory of experienced CD8 T cells before declining.

Age has previously been considered an important clinical factor in predicting irAEs in patients undergoing ICI treatment. For instance, some studies have suggested that age influences irAE frequency (36), whereas other analyses have reported minimal or no age-related differences (37, 38). However, despite GZMK was also reported as a hallmark of immune aging (20, 39), the expression of GZMK or GZMK-transitional signature score was not related with the chronological age of patients in our study.

In addition, patients in the AE group demonstrated an increased proportion of proliferating T cells, potentially amplifying clones with heightened reactivity. This proliferation not only underscores the robust immune activation induced by anti-PD-1 therapy but also aligns with increased instances of irAEs (13). In a prospective multi-center study of melanoma and NSCLC patients, ICI-treated individuals who developed irAEs exhibited an expansion of Ki-67+ T cell subsets (13). Such proliferation may lead to the expansion of clones capable of cross-reactivity with host tissues, thereby increasing the likelihood of developing irAEs.

Future work should define whether cytotoxic CD8 T cells in irAEs are autoreactive or tumor-specific via TCR clonality assays and antigen-specificity screens. Additionally, mechanistic studies exploring GZMK’s role in transitional cell function could unveil novel therapeutic targets to uncouple efficacy from toxicity. While our pilot study provides high-resolution insights, the findings require validation in a larger, multiple-time-point, independent cohort.

## Conclusion

In summary, our study underscores the delicate balance required to maximize the therapeutic efficacy of ICIs while minimizing adverse effects. By delineating the roles of cytotoxic, transitional, and proliferating CD8 T cells in irAE development, we provide new insights into immune dysregulation during anti-PD-1 therapy in acral melanoma patients. Mapping these cellular shifts and their functional consequences brings us closer to personalized strategies for predicting, preventing, and managing irAEs in melanoma. Such approaches are crucial for optimizing immunotherapeutic regimens and improving patient outcomes in this rapidly evolving field.

## Methods

### Patients and samples collection

Peripheral blood samples were collected from patients with melanoma admitted to the second affiliated hospital of Zhejiang University, School of Medicine. This study included patients with resectable stage II–III melanoma, as staged by the American Joint Committee on Cancer (AJCC, 8th edition). Six patients received adjuvant anti-PD-1 therapy, three of whom developed immune-related adverse events (irAEs), and two patients were under observation without treatment. All participants provided written informed consent, and the study protocol was approved by the local institutional ethics committee. Routine blood tests and clinical examinations were performed at regular intervals to monitor for early signs of irAEs. Peripheral blood mononuclear cells (PBMCs) were isolated from whole blood via density gradient centrifugation. For patients experiencing irAEs, PBMCs were collected within one week of irAE onset. For those without irAEs, samples were collected at a comparable time point following the initiation of anti-PD-1 therapy.

### Single-cell RNA sequencing

Cells were loaded onto the 10X Chromium Single Cell Platform (10X Genomics) at a concentration of 1,000 cells per μl (Single Cell 3′ library and Gel Bead Kit v.3) as described in the manufacturer’s protocol. Generation of gel beads in emulsion (GEMs), barcoding, GEM-RT clean-up, complementary DNA amplification and library construction were all performed as per the manufacturer’s protocol. Qubit was used for library quantification before pooling. The final library pool was sequenced on the Illumina HiSeq instrument using 150-base-pair paired-end reads.

### scRNA-Seq data processing, quality control, and analysis

Raw sequencing data in FASTQ format were processed using CellRanger (10x Genomics) to generate digital expression matrices. Low-quality barcodes and cells with fewer than 1,000 UMIs were excluded. Subsequent analyses were performed using the Seurat R package (v4.1.0) (40). Cells with fewer than 600 genes, more than 4,500 genes, or more than 10% mitochondria content were filtered out. Ribosomal genes were not filtered out pre-normalization. Data were normalized and integrated using the SCTransform method. Cell type annotation was performed via reference mapping using the MapQuery function according to Seurat v4 Vignettes (40). Then cells were split according to celltype.l1 for subpopulation analysis. Data were integrated for batch correction before further analysis. RunPCA, FindNeighbors, FindClusters, and RunUMAP were used to calculate the dimension-reduction coordinates for visualization and to perform unsupervised clustering. We used the first 30 principal components (PCs) for downstream analysis. For FindNeighbors and RunUMAP, a dim parameter of 1:30 was used. For FindClusters, a resolution parameter of 0.2 and 0.1 was used in all T cells and CD8 T cells sub-analysis, respectively. Differential expression analysis was conducted with the FindAllMarkers function. Gene Set Enrichment Analysis (GSEA) was performed using the clusterProfiler (v4.2.2) and enrichplot (1.14.2) packages. Pseudotime trajectory analysis was conducted with Monocle2 (v2.20.0). Prior to analysis, cells were downsampled to equal numbers across samples, and genes were ordered based on dispersional and empirical criteria. Dimensionality reduction for trajectory visualization was achieved using the DDRTree method.

### Statistical analysis

Cell population proportions were compared using non-parametric test (Mann-Whitney test) due to limited sample size. Bar charts were generated using GraphPad Prism (La Jolla, CA, USA). A p-value of less than 0.05 was considered statistically significant.

## Data Availability

The datasets presented in this study can be found in online repositories. The names of the repository/repositories and accession number(s) can be found below: https://ngdc.cncb.ac.cn/omix/preview/kOX6mQKk, OMIX011531.Processed data, including cell proportion tables and differentially expressed gene lists supporting the findings of this study, are provided in the **Supplementary Material**.
