# Supplementary material for: Single-cell profiling of PBMCS reveals an immune signature of irAEs in anti-PD-1-treated acral melanoma patients
Source: Front Immunol. 2026 Feb 26;17:1758205. doi: 10.3389/fimmu.2026.1758205 (PMC12979378; doi:10.3389/fimmu.2026.1758205)
Supplement: Supplementary file 1 [file DataSheet1.pdf]

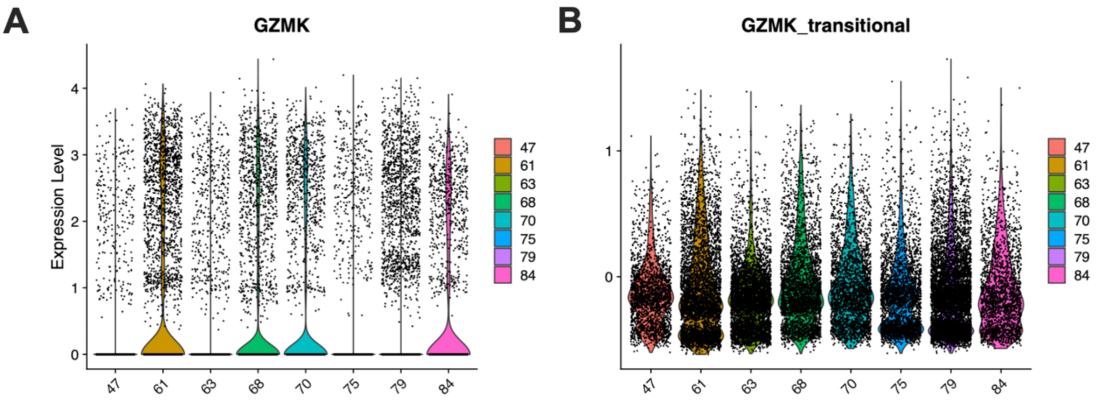


**Figure S1. The GZMK-transitional signature is not correlated with chronological age in T cells**

A-B. Violin plot showing the lack of correlation between the (A) expression level of GZMK, or (B) the GSVA score for the GZMK-transitional signature and the chronological age of each patient. Colored by samples, and ordered by age.
